# COVID-19: Rethinking the Lockdown Groupthink

**DOI:** 10.3389/fpubh.2021.625778

**Published:** 2021-02-26

**Authors:** Ari R. Joffe

**Affiliations:** ^1^Division of Critical Care Medicine, Department of Pediatrics, Stollery Children's Hospital, University of Alberta, Edmonton, AB, Canada; ^2^John Dossetor Health Ethics Center, University of Alberta, Edmonton, AB, Canada

**Keywords:** COVID-19, lockdowns, public health, cost-benefit analysis, groupthink

## Abstract

The Severe Acute Respiratory Syndrome Coronavirus 2 (SARS-CoV-2) has caused the Coronavirus Disease 2019 (COVID-19) worldwide pandemic in 2020. In response, most countries in the world implemented lockdowns, restricting their population's movements, work, education, gatherings, and general activities in attempt to “flatten the curve” of COVID-19 cases. The public health goal of lockdowns was to save the population from COVID-19 cases and deaths, and to prevent overwhelming health care systems with COVID-19 patients. In this narrative review I explain why I changed my mind about supporting lockdowns. The initial modeling predictions induced fear and crowd-effects (i.e., groupthink). Over time, important information emerged relevant to the modeling, including the lower infection fatality rate (median 0.23%), clarification of high-risk groups (specifically, those 70 years of age and older), lower herd immunity thresholds (likely 20–40% population immunity), and the difficult exit strategies. In addition, information emerged on significant collateral damage due to the response to the pandemic, adversely affecting many millions of people with poverty, food insecurity, loneliness, unemployment, school closures, and interrupted healthcare. Raw numbers of COVID-19 cases and deaths were difficult to interpret, and may be tempered by information placing the number of COVID-19 deaths in proper context and perspective relative to background rates. Considering this information, a cost-benefit analysis of the response to COVID-19 finds that lockdowns are far more harmful to public health (at least 5–10 times so in terms of wellbeing years) than COVID-19 can be. Controversies and objections about the main points made are considered and addressed. Progress in the response to COVID-19 depends on considering the trade-offs discussed here that determine the wellbeing of populations. I close with some suggestions for moving forward, including focused protection of those truly at high risk, opening of schools, and building back better with a economy.

## Introduction

The Severe Acute Respiratory Syndrome Coronavirus 2 (SARS-CoV-2) initially caused Coronavirus Disease 2019 (COVID-19) in China in December 2019, and has caused a worldwide pandemic in 2020. In response, most countries in the world implemented lockdowns, restricting their population's movements, work, education, gatherings, and general activities in attempt to “flatten the curve” of COVID-19 cases. Even now, as the so-called “second-wave” of COVID-19 cases is occurring, governments are considering and some implementing another lockdown to again “flatten the curve.” The public health goal of lockdowns is to save the population from COVID-19 cases and deaths, and to prevent overwhelming health care systems with COVID-19 patients. I was a strong proponent of lockdowns when the pandemic was first declared (1).

In this narrative review I explain why I changed my mind. First, I explain how the initial modeling predictions induced fear and crowd-effects (i.e., groupthink). Second, I summarize important information that has emerged relevant to the modeling. Third, I describe how reality started sinking in, with information on significant collateral damage from the response to the pandemic, and on the number of deaths in context. Fourth, I present a cost-benefit analysis of the response to COVID-19. I close with some suggestions for moving forward.

An important point must be emphasized. The COVID-19 pandemic has caused much morbidity and mortality. This morbidity and mortality have been, and continue to be, tragic. This narrative review aims to take these tragic outcomes of the pandemic seriously, and to also consider the tragic outcomes of the public health response to the pandemic. After all, lockdowns are a public health response undertaken with the goal of improving population health outcomes from the pandemic. Given adverse effects of lockdowns on many millions of people, with increased poverty, food insecurity, loneliness, unemployment, school closures, and interrupted healthcare, a cost-benefit analysis of lockdowns is necessary. We face terrible choices, but the response of lockdowns can be predicted to cause far more loss of population wellbeing than COVID-19 itself can.

## The Initial Predictions Induce Fear

### How It Started: Modeling

Early modeling made concerning predictions that induced fear (Table 1). Kissler et al. predicted the need for intermittent lockdowns occurring for a total of 75% of the time, even after July 2022, to avoid “overwhelming critical care capacity (page 866 in ref. 2)” (2–4). In their discussion they wrote that the response “is likely to have profoundly negative economic, social, and educational consequences… We do not take a position on the advisability of these scenarios given the economic burden… (page 868)” (2). On March 16, 2020, the Imperial College COVID-19 Response Team published modeling of the impact of non-pharmaceutical interventions (NPI) to reduce COVID-19 mortality and healthcare demand in the United States (US) and United Kingdom (UK) (5). They wrote that suppression “needs to be in force for the majority [>2/3 of the time] of the 2 years of the simulation (page 11),” without which there would be 510,000 deaths in Great Britain and 2.2 million deaths in the United States by mid-April, surpassing ICU demand by 30 times (5). In their discussion they wrote that “we do not consider the ethical or economic implications (page 4)… The social and economic effects of the measures which are needed to achieve this policy goal will be profound (page 16)…” (5). The Imperial College COVID-19 Response Team extended this to the global impact of the pandemic on March 26, 2020, and estimated that without lockdowns there would be “7.0 billion infections and 40 million deaths globally this year (page 1)” (6). In their discussion they wrote “we do not consider the wider social and economic costs of suppression, which will be high and may be disproportionately so in lower income settings (page 2)” (6). In a later publication, this group modeled that “across 11 countries [in Europe, since the beginning of the epidemic to May 4], 3.1 (2.8–3.5) million deaths have been averted owing to [NPI] interventions… (page 260)” (7). Another group similarly claimed that, in 5 countries (China, South Korea, Iran, France, US), NPIs “prevented or delayed [to April 6] on the order of 61 million confirmed cases (page 262)” (8).

**Table 1:** Initial modeling predictions that induced fear and crowd-effects.

**References**	**Statements and predictions from the modeling**
Kissler et al. (2–4)	“prolonged or intermittent social distancing may be necessary into 2022 [to avoid overwhelming critical care capacity]… expanded critical care capacity… would improve the success of intermittent distancing and hasten the acquisition of herd immunity”
	“projected that recurrent wintertime outbreaks of SARS-CoV-2 will probably occur after the initial, most severe pandemic wave [if immunity wanes over 40 weeks]”
	With a baseline reproductive number (Ro) 2.5, no seasonality to viral transmission, and the current intensive care capacity of the USA they projected the need for intermittent lockdowns occurring for a total of 75% of the time, even after July 2022.
Imperial College modeling of non-pharmaceutical interventions in USA and UK (5)	“suppression [effective reproductive number (Re)<1] will minimally require a combination of social distancing of the entire population, home isolation of cases and household quarantine of their family members. This may need to be supplemented by school and university closures… [and] Will need to be maintained until a vaccine becomes available.”
	“we show that intermittent social distancing – triggered by trends in disease surveillance – may allow interventions to be relaxed temporarily in relative short time windows….[Suppression] needs to be in force for the majority [>2/3 of the time] of the 2 years of the simulation.”
	The modeling assumed an IFR of 0.9%, hospitalization rate of 4.4%, and that 81% of the population would be infected before herd immunity, resulting in 510,000 deaths in Great Britain and 2.2 million deaths in the United States by mid-April, surpassing ICU demand by 30X, if lockdowns did not occur.
Imperial College modeling of non-pharmaceutical interventions globally (6)	“we estimate that in the absence of interventions, COVID-19 would have resulted in 7.0 billion infections and 40 million deaths globally this year… healthcare demand can only be kept within manageable levels through the rapid adoption of public health measures… to suppress transmission… sustained, then 38.7 million lives could be saved.”
	“[Suppression] will need to be maintained in some manner until vaccines or effective treatments become available.”
Imperial College estimate of lives saved so far in Europe (7)	Used a “model [that] calculates backwards [infections] from observed deaths… [and] relies on fixed estimates of some epidemiological parameters [Ro 3.8; attack rates in different age groups from 60-99%; infection fatality rate in different countries of 0.91–1.26%]….”
	Concluded that “we find, across 11 countries [in Europe], since the beginning of the epidemic [to May 4], 3,100,000 (2,800,000–3,500,000) deaths have been averted due to [NPI] interventions….”
Hsiang et al. (8)	In 5 countries [China, South Korea, Iran, France, US], using “reduced-form economic methods,” NPIs “prevented or delayed [to April 6] on the order of 62 million confirmed cases, corresponding to averting roughly 530 million total infections… we estimate that all policies combined slowed the average growth rate of infections [from 43%/day, a doubling time ~2 days] by −0.252 per day….”

### How It Took Off: Crowd Effects (Groupthink)

There ensued a contagion of fear and policies across the world (9–12). Social media spread a growing sense of panic (13). Popular media focused on absolute numbers of COVID-19 cases and deaths independent of context, with a “sheer one-sided focus (page 4)” on preventing infection (12). There was an appeal of group hysteria; “everyone got a break from their ambitions and other burdens carried in normal life,” and became united in crowds, which have a numbing effect (9). There was talk of “acting together against a common threat,” “about seeming to reduce risks of infection and deaths from this one particular disease, to the exclusion of all other health risks or other life concerns,” with virtue signaling to the crowd, of “something they love to hate and be seen to fight against” (9). A war effort analogy is apt, with the “unquestioning presumption that the cause is right, that the fight will be won, that naysayers and non-combatants [e.g., not wearing a mask] are basically traitors, and that there are technical solutions [e.g., vaccine and drugs] that will quickly overcome any apparent problem or collateral damage” (9). This was associated with a “disregard and disinterest on the part of individuals in the enormity of the collateral damage, either to their own kids, people in other countries, their own futures…” (9). The crisis was framed as a “war against an invisible enemy (page 5),” presenting the false choice between “lives and livelihood (page 5),” spreading fear and anxiety while ignoring the costs of the measures taken - this resulted in conformity and obedience (12, 13). There has been a strong positive association between new daily and total confirmed COVID-19 cases in a country and support for the heads of government, reflecting the “rally ‘round the flag’” effect [“the perception that one's group is under attack and hence unity is required to defend the group (page 25429)”] (14).

The NPIs spread to ~80% of OECD countries within a 2-week period in March 2020 (15). A main predictor of a country implementing NPIs was prior adoptions of a policy among spatially proximate countries, i.e., the number of earlier adopters in the same region (15). Variables not predicting adoption of NPIs included the number of cases or deaths, population >65 years old, or hospital beds per capita in the country (15). It seems we were all “stuck in this emotional elevation of COVID-19 deaths and suffering above everything else that could possibly matter” (16). There was the unquestioned assumption that “there were and are no alternatives to extreme measures implemented on entire populations with little consideration of cost and consequences [externalities] (page 477)” (10). Even now, how a country “performed” is measured by COVID-19 cases and deaths without denominators, without other causes of deaths considered, without considering overall population health trade-offs “that cannot be wished away” (e.g., the future of our children from lack of education and social interaction, and “changes to our wealth-generating capacity that has to pay for future policies”) (9), and without considering how sustainable current policies are [protection is temporary and leaves us susceptible; “there is no exit from the pandemic; there is only an exit from the response to it (page 479)” (10)].

All of this, even though in October 2019 the WHO published that for any future Influenza pandemic: travel-related measures are “unlikely to be successful… are likely to have prohibitive economic consequences (page 2)”; “[measures] not recommended in any circumstances: contact tracing, quarantine of exposed individuals, border closure (page 3)”; social distancing measures (closures of workplace, avoiding crowding and closing public areas) “can be highly disruptive, and the cost of these measures must be weighed against their potential impact (page 4)”; and “border closures may be considered only by small island nations in severe pandemics… but must be weighed against potentially serious economic consequences (page 4)” (17). Referring to the 2009 influenza pandemic, Bonneux and Van Damme wrote that “the culture of fear” meant that “worst-case thinking replaced balanced risk assessment” on the part of influenza “experts” (page 539) (18). But “the modern disease expert knows a lot about the disease in question, but does not necessarily know much about general public health, health economics, health policy, or public policy, which are much more about priority setting and hence resource allocation between competing priorities [because resources are limited, wise allocation saves lives]” (19).

Some of this crowd effect is related to cognitive biases, “the triumph of deeply human instincts over optimal policy” (page 303 in ref 21) (20–22). Identifiable lives bias included the identifiable victim effect (we ignore hidden “statistical” deaths reported at the population level), and identifiable cause effect (we prioritize efforts to save lives from a known cause even if more lives would be saved through alternative responses). Present bias made us prefer immediate benefits to even larger benefits in the future (steps that would prevent more deaths over the longer term are less attractive) (20–22). The proximity and vividness of COVID-19 cases (i.e., availability and picture superiority bias), and anchoring bias (we adhere to our initial hypothesis, and disregard evidence that disproves our favorite theory) affected our reasoning (21, 23). Superstitious bias, that action is better than non-action even when evidence is lacking, reduced anxiety (12). Escalation of commitment bias, investing more resources into a set course of action even in the face of evidence there are better options, made us stand by prior decisions (24). We need to take an “effortful pause,” reflecting on aspects of the pandemic that do not fit with our first impressions (25). The groupthink (the tendency for groups to let the desire for harmony and conformity prevail, resulting in dysfunctional decision-making processes and becoming less willing to alter their course of action once they settle on it) needs to be replaced by deliberative consideration of all the relevant information (24).

## Important New Information Emerging

### The Infection Fatality Rate (IFR)

Based on seroprevalence data as of September 9, 2020, including 82 estimates from across 51 locations in the world, Ioannidis found that the median corrected IFR was 0.23% (range 0.00–1.54%) (26). Among those <70 years old the median crude and corrected IFR was 0.05% (range 0.00–0.31%). He estimated that for those <45 years old the IFR was almost 0%, 45–70 years old approximately 0.05–0.30%, and ≥70 years old ≥ 1%, rising to up to 25% for some frail older people in nursing homes (27). He estimated that at that point there were likely 150–300 million infections that had occurred in the world, not the reported 13 million, most being asymptomatic or mildly symptomatic (26, 27). The WHO recently estimated that about 10% of the global population may have been already infected, which, with a world population of 7.8 billion, and 1.16 million deaths, would make a rough approximation of IFR as 0.15% (28).

Even these numbers are most likely a large *over-estimate* of the IFR. First, in serosurveys the vulnerable (e.g., homeless, imprisoned, institutionalized, disadvantaged people), who have higher COVID-19 incidence, are more difficult to recruit. Second, there is likely a healthy volunteer bias in serosurvey studies. Third, and most importantly, there is a lack of sensitivity of serology (29–34). Many reports now document there is often a rapid loss of antibody in COVID-19 patients that were less severely ill (29–36). Moreover, at least 10% of COVID-19 patients never seroconvert, and many more may only develop a mucosal IgA response (37, 38), or only a T-cell response (which may be the case in up to 50% of mild infections) (39, 40). Finally, most data come from unusual epicenters where infection finds its way into killing predominantly older citizens in nursing homes and hospitals (26), and where “[in Italy, Spain, France] an underfunded, understaffed, overstretched and increasingly privatized and fractured healthcare system contribute to higher mortality rates… [Lombardy] has long been an experimental site for healthcare privatization (page 474–475)” (10). With “precise non-pharmacological measures that selectively try to protect high-risk vulnerable populations and settings, the IFR may be brought even lower (page 10)” (26).

A serology-informed estimate of the IFR in Geneva, Switzerland put the IFR at: age 5–9 years 0.0016% (95% Credible Interval, CrI 0, 0.019), 10–19 years 0.00032% (95% CrI 0, 0.0033), 20–49 years 0.0092% (95% CrI 0.0042, 0.016), 50–64 years 0.14% (95% CrI 0.096, 0.19), and age 65+ outside of assisted care facilities 2.7% (95% CrI 1.6, 4.6), for an overall population IFR 0.32% (95% CrI 0.17, 0.56) (41). Similarly, a large study from France found an inflection point in IFR around the age of 70 years (see their Figure 2D) (42).

### High-Risk Groups

Ioannidis et al. analyzed reported deaths from epicenters, in 14 countries and 13 states in the United States, to June 17, 2020 (43). They found that in those age <65 years the relative risk of death was 30–100X lower in Europe and Canada, and 16–52X lower in the USA, compared to those ≥65 years old (43). They estimated that those age 40–65 years old have double the risk of the overall <65 year-old group, and females have 2X lower risk than males (43). This is compatible with a steep inflection point in the IFR around the age of 70 years old. Older adults in nursing homes accounted for at least half of the COVID-19 deaths in Europe and North America, and over 80% in Canada (44, 45). In nursing homes the usual median survival is ~2.2 years, with a yearly mortality rate >30%, even without COVID-19 (46). Outbreaks of the seasonal respiratory coronavirus in adults living in long-term care facilities are common, with case-fatality rates of 8% (47). Ioannidis et al. estimated that the average daily risk of COVID-19 death for an individual <65 years old was equivalent to the risk from driving between 12–82 miles/day during the pandemic period, higher in the UK and 8 states (106–483 miles/day), and only 14 miles/day in Canada (43).

By far the most important risk factor is older age (41–43). There is an approximately 1,000-fold difference in death risk for people >80 years old vs. children (43). In the largest observational study I am aware of, the OpenSAFELY population in the UK, including over 17 million people with 10,900 COVID-19 deaths, compared to those age 50–59 years old, the Hazard Ratio for death from COVID-19 ranged from 0.06 for those age 18–39 years, to >10 for those age >80 years (48). In comparison, even important co-morbidities such as severe obesity, uncontrolled diabetes, recent cancer, chronic respiratory or cardiac or kidney disease, and stroke or dementia rarely had HR approaching ≥2 (48). Those co-morbidities with HR>2, including hematological malignancy, severe chronic kidney disease, and organ transplant, affected only 0.3, 0.5, and 0.4% of the total population (48).

A rapid systematic review found that only age had a “consistent and high strength association with hospitalization and death from COVID-19… strongest in people older than 65 years…(page 1)” (49). Other risk groups for mortality had either a low-moderate effect (obesity, diabetes mellites, male biological sex, ethnicity, hypertension, cardiovascular disease, COPD, asthma, kidney disease, cancer) and/or were inconsistently found to have an effect in the literature (obesity, diabetes mellites, pregnancy, ethnicity, hypertension, cardiovascular disease, COPD, kidney disease) (49). Even with these risk factors, the absolute risk may still be low, given the overall IFR in the population at that age.

### Objection: Is This Age Discrimination?

An objection may be that singling out older people as high risk is age discrimination. This is false on two counts. First, pointing out the truly high-risk group is in older people is only emphasizing that this is the group that requires protection from severe COVID-19 outcomes. Second, as Singer has pointed out, “what medical treatment does, if successful, is prolong lives. Successfully treating a disease that kills children and young adults is, other things being equal, likely to lead to a greater prolongation, and thus do more good, than successfully treating a disease that kills people in the 70's, 80's, and 90's” (50). In fact, when we try to stay healthy “what we are trying to do is to live as long as we can, compatibly with having a positive quality of life for the years that remain to us. If life is a good, then, other things being equal, it is better to have more of it rather than less” (50). We should count every quality adjusted life year equally, whether it is in the life of a teenager or a 90-year old (50, 51). This was also the conclusion of “The Fair Priority Model” for global vaccine allocation, prioritizing preventing premature death using a standard expected years of life lost metric (52).

Veil of ignorance reasoning is a widely respected and transparent standard for adjudicating claims of fairness. A fair distribution of resources is said to be one that people would choose out of self-interest, without knowing whom among those affected they will be: what would I want if I didn't know who I was going to be? In an experimental study participants were asked to decide whether to give the last available ventilator in their hospital to the 65 year old who arrived first and is already being prepped for the ventilator, or the 25 year old who arrived moments later, assuming whoever is saved will live to age 80 years old. In the veil of ignorance condition, the participant was asked to “imagine that you have a 50% chance of being the older patient, and 50% the younger” (53). Asked if “it is morally acceptable to give the last ventilator to the younger patient,” 67% in the veil of ignorance condition vs. 53% in control answered “yes” (odds ratio 1.69; 95% Confidence Interval 1.12, 2.57); compared to younger age participants (18–30 years), older participants (odds ratio 3.98) and middle age participants (odds ratio 2.02) were more likely to agree (53). Asked if “you want the doctor to give the ventilator to the younger patient,” 77% answered “yes,” maximizing the number of life-years saved rather than the number of lives saved (53). Thus, veil of ignorance reasoning resulted in the participants counting every quality adjusted life year equally, whether it is in the life of a 25-year old or a 65-year old.

### The Herd Immunity Threshold

The classical herd immunity level is calculated based on the basic reproduction number (Ro) as (1–1/Ro), and is the proportion of the population that must be immune to a virus before the effective reproduction number (Re) is <1, and thus the virus cannot perpetuate itself in the population. This calculation assumes a homogeneously mixing population, where all are equally susceptible and infectious. For Ro 2.5, the threshold is ~60% of the population. However, the assumption is not valid, as there is heterogeneity in social mixing and connectivity, with higher and lower levels of activity and contacts. One model incorporating heterogeneity of social mixing found the threshold, for Ro 2.5, to be 43%, and likely lower as other heterogeneity in the population was not modeled (e.g., sizes of households, attending school or big workplaces, metropolitan vs. rural location, protecting older people, etc.) (54). A model that incorporated variation in connectivity compatible with other infectious diseases found that for Ro 3, the threshold is 10–25% of the population developing immunity (55). Another model that “fit epidemiological models with inbuilt distributions of susceptibility or exposure to SARS-CoV-2 outbreaks” calculated “herd immunity thresholds around 10-20% [because]… immunity induced by infection… [contrary to random vaccination] is naturally selective (page 2)” (56). In support of this heterogeneity, it is now known that there is overdispersion of transmission of SARS-CoV-2, with 80% of secondary infections arising from just ~10% of infected people (57–59).

### Objection: Consider Sweden

It has been claimed that Sweden's strategy of achieving herd immunity failed, with excess deaths and a suffering economy. However, that is not clear. First, cases and deaths fell consistently in later July/August, with deaths continuing at a very low level into October despite no lockdown (60). Second, serosurveys in mid-July found 14.4% of the population may be seropositive; thus, with 5,761 deaths as of August 1, in a population of 10.23 million, the crude IFR may have been 0.39%, and even lower considering the sensitivity of serology discussed above (61). Early on, Sweden did not adequately protect those in nursing homes, a failing that also inflates the IFR (62). The excess all-cause mortality per 100,000 up to July 25, 2020 in Sweden was 50.8, lower than in England and Wales, Spain, Italy, Scotland, Belgium, Netherlands, France, and the US (62, 63). Third, in a globalized world, with entangled webs of supply, demand, and beliefs, what we do here will devastate people not just here, but also elsewhere and everywhere (64). Compared to Denmark, with an economy heavily dependent on pharmaceuticals, Sweden's recession looks bad. However, compared to the European Union, Sweden looks good; the European Commission forecasts a better 2020 economic result for Sweden (GDP−5.3%) than many other comparable European countries (e.g., France−10.6%, Finland−6.3%, Austria−7.1%, Germany−6.3%, Netherlands−6.8%, Italy−11.2%, Denmark−5.2%) (65).

### The Exit Strategy

Herd immunity appears to be the only exit from the response to COVID-19. This can be achieved naturally, or through vaccine. For the reasons given here, it is very possible that the lockdowns are only delaying the inevitable.

There are problems with the natural herd immunity approach involving the currently projected and implemented waves of lockdowns. First, this will take years to occur, causing economic and social devastation. This also assumes immunity is long-lasting such that cycles of shutting down can be successful over 2 or 3 years, and without which it is more likely COVID-19 will be an annual occurrence (2). Second, the less devastating test-trace-isolation/quarantine strategy seems not feasible. In the United States it was estimated that there would be a need to train an extra 100,000 public health workers, and to do >5 million SARS-CoV-2 tests per day, necessitating the building of many new very large testing factories (66). Countries would still need to keep borders closed and maintain physical distancing (e.g., no large events) in order to make contact tracing feasible; this would be for years, during which people may become very reluctant to be tested. Modeling suggests that to be successful, because asymptomatic and pre-symptomatic individuals may account for 48–62% of transmission (even in nursing home residents) (67), contact tracing and quarantine would have to occur within 0.5 days for >75% of contacts, necessitating mobile app technology that has its own feasibility and ethical problems (68–70).

Vaccine induced herd immunity involves many assumptions. First, there will be the discovery of an effective and safe vaccine that does not cause antibody-dependent (or other immune) enhancement; this, even though the problem in severe COVID-19 may be the host response, especially in older people and children (71–73). Second, the immune response will be durable, not last for only months, and have little immunosenescence (reduced response to vaccine with rapid decline of antibody levels) in older people (72, 74). Third, that mass production and delivery of the vaccine will occur very soon, and be done equitably to all humans on Earth; otherwise, there is the risk of conflict, war, and terrorism in response to gross inequity in vaccine distribution (52). In response to the 2009 pandemic of H1N1 Influenza the United States achieved a weekly vaccination rate of only 1% of the population (72). Vaccine refusers may include 30% of the population in North America and globally (72, 75), and if they have “increased contact rates relative to the rest of the population, vaccination alone may not be able to prevent an outbreak (page 817)” (72). There is already competition among high income countries, and likely crowding out of low-income countries that represent about half of the human population (76). The only globally eradicated human disease is smallpox, which took “30 years to achieve,” and the “fastest historical development of a [new] vaccine was 4 years (Merck: mumps), while most take 10 years (page 2)” (77).

## Reality Sinking In

### Iatrogenic Collateral Harms: Lockdown as a “Drug” With Dangerous Side-Effects When Its Use Is Prolonged

The COVID-19 response has threatened to make, and likely has already made, several Sustainable Development Goals for the most vulnerable among us in low-income countries out of reach (78–82). The numbers involved are staggering, and in the many millions (Table 2). The response has had major detrimental effects on childhood vaccination programs, education, sexual and reproductive health services, food security, poverty, maternal and under five mortality, and infectious disease mortality (78–93). The effect on child and adolescent health will “set the stage for both individual prosperity and the future human capital of all societies (page 2)” (94). The destabilizing effects may lead to chaotic events (e.g., riots, wars, revolutions) (95, 96).

**Table 2:** Some effects of the COVID-19 response that put sustainable development goals out of reach (78–93).

**Sustainable development goal**	**Effect of COVID-19 response: some details**
Childhood vaccination	Programs stalled in 70 countries (Measles, Diphtheria, Cholera, Polio)
Education	School closures: 90% of students (1.57 Billion) kept out of school -Early primary grades are most vulnerable, with effects into adulthood: effects on outcomes of intelligence, teen pregnancy, illicit drug use, graduation rates, employment rates and earnings, arrest rates, hypertension, diabetes mellites, depression -Not just education affected: school closures have effects on food insecurity, loss of a place of safety, less physical activity, lost social interactions, lost support services for developmental difficulties, economic effects on families
Sexual and reproductive health services	Lack of access: estimated ~2.7 Million extra unsafe abortions For every 3 months of lockdown: estimated 2 Million more lack access to contraception, and over 6 months, 7 Million additional unintended pregnancies
Food security	Hunger pandemic: undernourished estimated to increase 83–132 Million (>225,000/day; an 82% increase) -from disrupted food supply chains (labor mobility, food transport, planting seasons) and access to food (loss of jobs and incomes, price increases)
End poverty	Extreme poverty (living on < US$1.90/day): estimated to increase >70 Million -Lost “ladders of opportunity” and social determinants of health
Reduce maternal and U5M	Estimated increase of 1.16 Million children (U5M) and 56,700 maternal deaths, if essential RMNCH services are disrupted (coverage reduction 39–52%) for 6 months in 118 LMIC mostly (~60%) due to affected childhood interventions (wasting, antibiotics, ORS for diarrhea); and childbirth interventions (uterotonics, antibiotics, anticonvulsants, clean birth)
Infectious Disease Mortality	Tuberculosis: in moderate and severe scenario, projected excess deaths (mostly from reduced timely diagnosis and treatment) 342,000–1.36 Million over 5 years (an increase of 4–16%) Malaria: in moderate and severe scenario, projected excess deaths (mostly from delayed net campaigns and treatment) 203,000 to 415,000 over 1 year (an increase of 52–107%, with most deaths in children <5 yo). HIV: in moderate projected excess deaths (mostly due to access to antiretrovirals) 296,000 (range 229,000–420,000) in Sub-Saharan Africa over 1 year (an increase of 63%). Also would increase mother to child transmission by 1.6 times.

*LMIC, low- and middle-income countries; ORS, oral rehydration solution; RMNCH, Reproductive Maternal Newborn and Child Health; U5M, under 5 mortality*.

In high-income countries, the collateral damage has also been staggering (Table 3), affecting visits to emergency departments and primary care for acute (e.g., myocardial infarction, stroke) and “non-urgent” (“elective” surgery, and cancer diagnosis and treatment) conditions, intimate partner violence, deaths of despair, and mental health (12, 97–112). Of excess deaths occurring during the pandemic in high-income countries, 20–50% are not due to COVID-19 (62, 113–115). There was an unexplained 83% increase of 10,000 excess deaths from dementia in England/Wales in April, and an increase in non-COVID-19 Alzheimer disease/dementia deaths in the US, attributed to lack of social contact causing a deterioration in health and wellbeing of these patients (115, 116).

**Table 3:** Some effects of the COVID-19 response on public health in mostly high-income countries (97–119).

**Effect of COVID-19 response**	**Some details**
Delayed/avoided/disrupted medical care	Visits to emergency departments for myocardial infarction or stroke declined in USA by ≥20–48%
	Delayed cancer care and “non-urgent” procedures - weekly presentations with cancer diagnoses down 46% in USA and UK - 90% reduction in non-cancer surgeries in Ontario in March/April - surgery backlog in Ontario March 15 to June 13: 148,000; clearance time estimated to take 84 weeks - in Canada at least $1.3 billion additional funding is required to return to pre-pandemic wait times for six procedures (CABG, cataract surgeries, hip and knee replacements, MRI and CT scans) within 1 year
	Of excess deaths in high-income countries during pandemic, 20-50% are *not* from COVID-19
	Unexplained 83% increase (10,000 excess) deaths from dementia in England/Wales in April (lack of social contact causing a deterioration in health and wellbeing)
Violence against women (household stress; disrupted livelihoods, social/protective networks, support services)	Intimate Partner Violence: estimated effect from 3 months lockdown is 20% increase (>15 Million additional cases) Female Genital Mutilation: 2 Million more cases over next decade Child Marriages: 13 Million more cases over next decade Increased police reports (France, UK, Ontario) and support line calls (China, Italy, Spain, Vancouver, Alberta) by 20–50%
Deaths of despair (related to unemployment, and due to drugs, alcohol, and suicide)	In USA alone: 68,000 (from 27,000–154,000) suicide deaths predicted
	Mental Health effects of 3 months (suicide, depression, alcohol use disorder, childhood trauma due to domestic violence, changes in marital status, social isolation): Years of Life Lost in USA 67.58 Million, Canada 7.79 Million, UK 13.62 Million, etc. Surge in Canada in opioid deaths (by 40–50%), alcohol consumption (by 19%), cannabis use (by 8%), tobacco smoking (by 4%), and suicidal thoughts.

COVID-19 “Is a disease of inequality and it also creates even more inequality (page 3)” (95). Unequal structural determinants of health meant that disadvantaged minorities have experienced a greater toll from the COVID-19 “Great Lockdown” (117), with contributors including lower income (e.g., economic and job insecurity), homelessness or crowding at home (and in transportation), worse health care (and pre-existing health disparities), and inability to work from home (e.g., for essential, manual, and temporary workers) (45, 95, 118, 119). COVID-19 policing has involved “racial profiling and violence, crippling punishments for those living in poverty, and criminalization of mental health (page E1219)” (120). Refugees are particularly vulnerable, undertaking “arguably the most essential form of travel… with little access to water, space or health care (page E1219)” (120). The effect on the health of women and girls is particularly severe, disproportionately affecting sexual and reproductive health services, income, and safety (121, 122).

### Numbers in Context

Numbers without denominators and without context are deceiving. Some data in this section may put the COVID-19 pandemic numbers in perspective.

Assuming all deaths *with* COVID-19 are deaths *from* COVID-19, in the USA as of August 22, 2020, COVID-19 was the cause of 9.24% of overall deaths; this means that >90% of deaths are not a focus of our attention (Supplementary Table 1) (123). Similarly, in Canada, COVID-19 was the cause of 5.96% of estimated deaths over the first 6 months of 2020, again meaning >94% of deaths are not a focus of our attention, and not being reported daily in the press as are COVID-19 deaths (Supplementary Table 2) (124, 125). By late-November these relative numbers had changed little (Supplementary Table 3) (123–125). A similar analysis in the UK found that, during 16 weeks of the pandemic, the risk of death was “equivalent to experiencing around 5 weeks extra ‘normal’ risk for those over [age] 55, decreasing steadily with age, to just 2 extra days for schoolchildren… [and in those] over 55 who are [detected as] infected with COVID-19, the additional risk of dying is slightly more than the “normal” risk of death from all other causes over one year (page 6)” (126).

Across the world in 2019 there were 58,394,000 deaths, >4.87 million deaths/month and >159,983 deaths/day; COVID-19 deaths are shown relative to these underlying deaths in Table 4 and Supplementary Table 3 (127, 128). The number of deaths is highly unequal, with far more deaths at earlier ages in low-income countries and Sub-Saharan Africa (127). If all countries were to achieve the Sustainable Development Goal of Under 5 Mortality Rate <25 deaths/1,000 by 2030, from the year 2015 this would avert 12.8 million deaths (129). From 2000–2017, if all units had an Under 5 Mortality Rate that matched the best performing unit in each respective country, this would have averted 58% of deaths in those under 5 years, that is, 71.8 (68.5–74.9) million deaths (130). A realistic projection was that if the pandemic takes 5 years for “full cycling,” 60% of the global population is infected, and the IFR is 0.19%, COVID-19 will account for 2.9% of global deaths. If only 10% of the high-risk population are infected, COVID-19 will account for 0.6% of global deaths over 5-years (95).

**Table 4:** World mortality data 2019, with COVID-19 mortality to Sept 4 in 2020 for comparison (127, 128).

**Region**	**Annual deaths in thousands (per day)**	**Infant mortality Rate/1,000**	**Under 5 yo mortality Rate/1,000 (% of deaths)**	**Age 15–60 mortality Rate/1,000 (% of deaths)**	**Age 65+ (% of deaths)**
World	58,394 (160)	28	38 (10%)	140 (32%)	(57%)
**COVID-19 on Sept 4, 2020**	**865 (3.5)**	**(0%)**	**(0.06%)**	**(26%)**	**(74%)**
High-income	11,161	4	5 (1%)	81 (19%)	(80%)
Middle-income	41,551	27	35 (9%)	144 (36%)	(55%)
Low-income	5,665	46	68 (31%)	234 (42%)	(27%)
Sub-Saharan Africa	9,052	49	74 (31%)	281 (46%)	(23%)
Canada	291	4	5 (1%)	62 (17%)	(82%)

*Effect of COVID-19 is in bold for emphasis*.

Some causes of death in the world are given in Table 5; COVID-19 deaths (~3,500/day up to September 4, 2020) are also shown (131–143). For example, there are an estimated 4,110 deaths/day from Tuberculosis (133), 3,699 deaths/day from motor vehicle collisions (131), 21,918 deaths/day due to use of tobacco (132), >3,400 deaths/day from Under 5 cases of pneumonia or diarrhea (137, 138), and 30,137 deaths per day from dietary risk factors (139). The WHO has estimated that if all people would adopt a vegan diet this would avert 13.7 M (95% CI 7.9, 19.4) deaths by 2030 (84). Some of these deaths are preventable if we were to take appropriate action, and some we as a society have decided we are willing to accept in trade-off for our freedom and wellbeing.

**Table 5:** Selected causes of death in the world, with deaths per year and day, compared to COVID-19 in 2020 (131–143).

**Cause of death**	**Deaths/year (/day)**	**Case fatality rate**	**Age group predominant**
**COVID-19 on Sept 4, 2020**	**864,618 (3,500)**	**0.24%**	**≥65–70 years old**
Malaria	405,000 (1,110)	0.2%	Children
Tuberculosis	1,500,000 (4,110)	<15%	–
Measles	140,000 (384)	1.46%	Children
Influenza	389,213 (range 294–518 K)^a^	0.01–0.02% for pH1N1	Children 34,800 (13–97 K), and ≥65 years old. Respiratory deaths only
HIV	690,000 (1,890)	–	Access to treatment for 67%
Motor Vehicle Collisions	1,350,000 (3,699)	–	Young 5–29 years old, mostly in Low- to Middle-Income Countries
Tobacco	>8,000,000 (21,918)	–	–
Childhood (U5M) pneumonia	808,920 (2,216)	–	<5 years old
Childhood (U5M) diarrhea	533,768 (1,462)	0.08% U5M	<5 years old
Dietary risk factors	11,000,000 (30,137)	–	–

a *The 1957–1959 Influenza pandemic, when the world population was 2.87 billion, was estimated to cause 4 deaths/10,000 population totaling 1.1 million excess deaths due to respiratory disease, with the greatest excess mortality in school-aged children and young adults. If COVID-19 is of similar severity, given the world population of 7.8 billion, we would expect ~3 Million deaths, mostly in older people (143)*.

*K, thousands; U5M, under 5 mortality. Effect of COVID-19 in bold for emphasis*.

## An Informed Cost-Benefit Analysis of Lockdowns

### The Corona Dilemma

**Figure 1:**
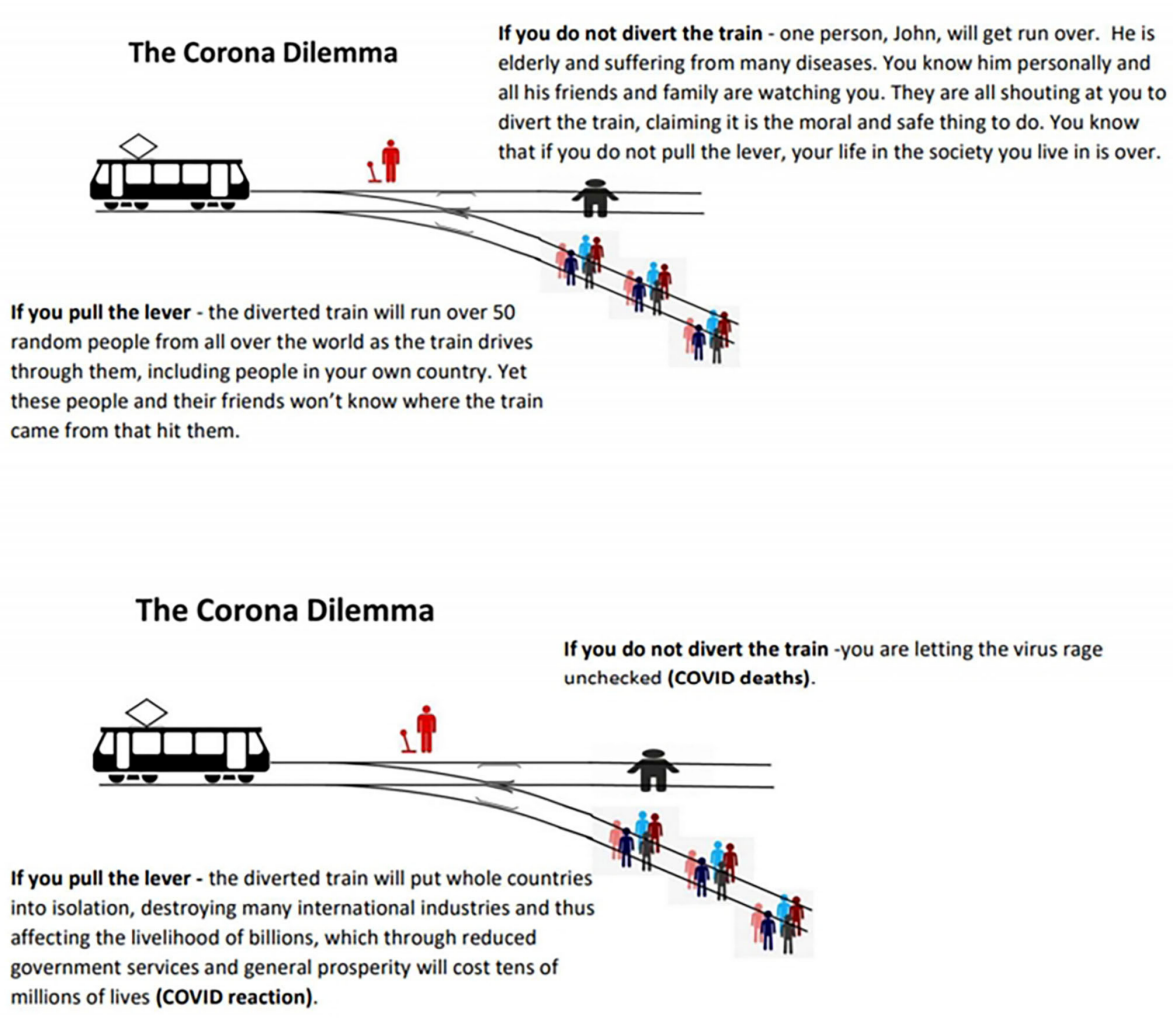
**(A)** The Trolley Dilemma using numbers compatible with the Corona Dilemma. Modified with permission from Frijters (144). **(B)** The Corona Dilemma choices explicitly explained. Modified with permission from Frijters (144).

The economist Paul Frijters has asked us to consider “The Corona Dilemma” (Figures 1A,B) modeled after the so-called “Trolley Problem” in philosophy (144). He asks us to imagine “you are the decision maker who can pull the lever on the train tracks to avoid the coming train from going straight” (144). Our options are to divert the train or not. “If you do not divert the train – you are letting the virus rage unchecked [i.e., COVID-19 deaths]” (144). On the other hand, “if you pull the lever – the diverted train will put whole countries into isolation, destroying many international industries and thus affecting the livelihood of billions, which through reduced government services and general prosperity will cost tens of millions of lives [i.e., COVID-19 reaction]” (144). The world pulled the lever, and the unintended health consequences of these measures did not play a part in modeling or policy.

### Cost-Benefit Analysis

Medical and Public Health experts are not expert in this type of analysis (18, 19). Health resources are finite. We all take health risks to ensure a better future for ourselves, family, children, and society. “Wellbeing of the population is the ultimate goal of government (page 6 in ref 146)” (145, 146). To compare outcomes of policies we need a common single metric of measurement to weigh trade-offs and make rational decisions. The goal is to maximize the sum of years lived by the population (52), weighted by the health quality of those years (i.e., Quality Adjusted Life Years, QALY) or the wellbeing quality of those years (i.e., Wellbeing Years, WELLBY). The QALY misses some important things that are valued by individuals, including joy, status, and things that give fulfillment like jobs. The WELLBY measures the value of anything that makes life enjoyable, and captures almost everything that is important to people. It is measured by life satisfaction, asking “overall, how satisfied are you with your life nowadays?” and rated on a Likert Scale from 0 (“not at all”) to 10 (“completely”); the usual healthy level is “8,” and those indifferent between living on or not at all score “2” – 1 regular year of happy life (1 QALY) is worth 6 WELLBY (145, 146). Despite some limitations, cost and benefit should be measured in terms of human welfare in the form of length, quality, and wellbeing of lives, and “to make no assessment is just to make policy in a vacuum (page 9)” (147).

First, consider the benefits of lockdown, preventing COVID-19 deaths. Using the age distribution of deaths and comorbidities, in the UK the average person who died due to COVID-19 had 3–5 healthy years left to live; that is, 3–5 QALY, or 18–30 WELLBY (95, 144, 147). This number was even lower in Italy (144). We can calculate the QALY and WELLBY that lockdowns “saved” by multiplying the following factors:

50% of the population will be infected to achieve herd immunity ×
0.3% IFR in the population ×
7.8 Billion people in the world population
= 11.7 million deaths ×
5 QALY lost per death = 58.5 million QALY ×
6 WELLBY per QALY = 360 million WELLBY.

The number is likely much lower than this for several reasons: it is likely <40% to achieve herd immunity, the IFR is likely <0.24%, some deaths would occur even with lockdowns (that might prevent at most 70% of deaths; in Sweden it was estimated lockdown could have prevented one-third of deaths) (148), with focus on retirement and nursing homes we might avoid many of the excess deaths, and we cannot stay locked down forever [if no “exit strategy” exists, then lockdown is not really a “strategy” (10)]. A more realistic number is at least 2X lower, well fewer than 5.2 million deaths “saved.” It is also worth mentioning that the efficacy of lockdown has been questioned in several studies, reducing the benefit of lockdown potentially markedly further (Supplementary Table 4) (149–155).

Second, consider the costs of lockdown (144, 156–158). An important point must be made here. We are not comparing COVID-19 deaths vs. economy as prosperity. Rather, it is COVID-19 deaths vs. recession deaths – it's lives vs. lives, as the economy is about lives. “It's horrible either way… [we're] advocating for the least people to die as possible” (159).

**Figure 2:**
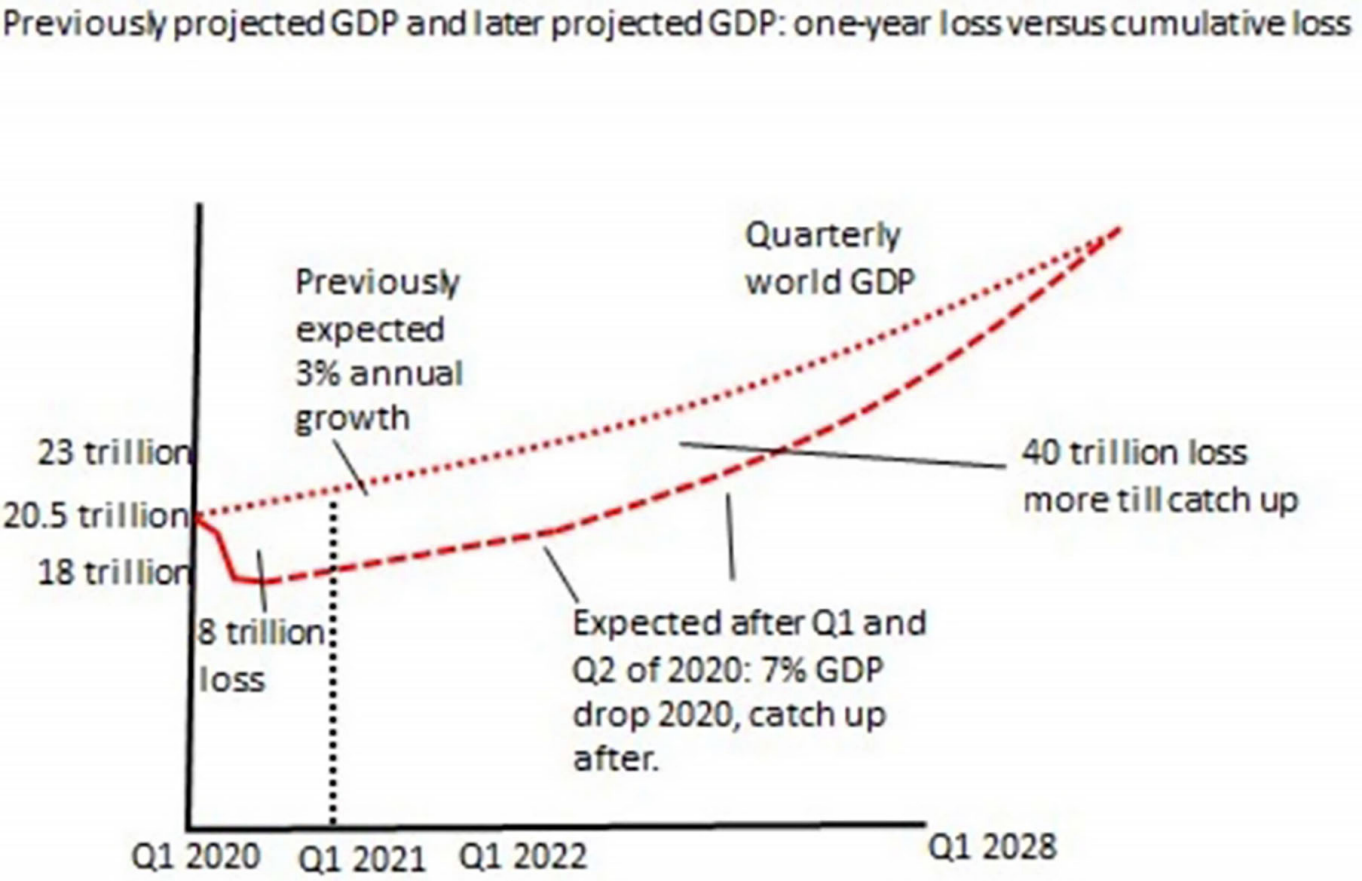
Explanation of how acute GDP loss of 6-7% will accumulate over the decade to a loss of at least US$50 trillion. Reproduced with permission from Frijters (Personal Communication).

Expected costs of the recession in lives can be calculated based on two methods. One uses historical evidence of a strong long-run relation between government spending (economic development) and life expectancy (144, 156–158). Government expenditures on healthcare, education, roads, sanitation, housing, nutrition, vaccines, safety, social security nets, clean energy, and other services determines the population wellbeing and life-expectancy (144). If the public system is forced to spend less money on our children's future, there are statistical lives lost (people will die in the years to come). The social determinants of health, including conditions of early childhood, education, work, social circumstances of elders, community resilience (transportation, housing, security), and fairness (economic security) determine lifespan (160). As a general rule, US$10K/year GDP buys an additional 10 years of life, so in a life of 75 years, US$10K/year X 75 years = US$750K buys 10 years in life expectancy = US$75K/QALY (144, 156–158). This is a maximum cost; in India US$25K/QALY is appropriate (most effect occurs for vulnerable and marginalized groups) (144). The other method is based on government numbers that are used to estimate how much health and life expenditures buy. Since the lockdown is a government public health policy, “it is saving of lives which is what the lockdown was for… we are treating decisions on how to face COVID-19 in the same way as decisions… are made about resources to apply to the treatment of cancer, heart disease, dementia, and diabetes (page 11)” (147). Based on research on how costly it is to save people from illness (how government services maintain health), in the UK it is US$20K/QALY, and using consumer willingness to pay it is US$80K/QALY (144–146). This again is a maximum cost, as this is for Western countries, who are at least 3X wealthier than the average country in the world; you can save a life in poor countries with US$2-3K, and lives are saved more cheaply with the first few billions spent (144, 161). It is estimated that in 2020–2021 the world economy will shrink by at least US$8-9 trillion (about 6% of GDP), and this will take many years to recover (Figure 2) (144, 156, 157, 162, 163). The loss in terms of GDP will be “easily US$50 trillion over the coming decade” (144, 156), with lockdowns ordering businesses and workplaces to stop functioning, ports closed, business bankruptcies, and resultant disrupted supply and demand chains (64, 164, 165). We can calculate what the recession resulting fromlockdowns “cost” as follows:

US$50 trillion loss in GDP ×
40% of GDP as government expenditure ÷
US$100K/QALY = 200 million QALY ×
6 WELLBY per QALY = 1.2 billion WELLBY.

This is an underestimate, and the actual figure is likely at least 12X higher for several reasons: the number US$100K/QALY was used when it is far less than this for half the world population residing in low-income countries and may be much lower even in high-income countries, and a conservative estimate of world GDP loss during the pandemic was used, particularly if there is another prolonged period of lockdown.

Another cost of lockdown is the loneliness and anxiety effect on individuals. It is estimated that loneliness from isolation costs 0.5 WELLBY/person/year (145, 146). If lockdowns last for 2 months to 4 billion people, this results in a cost of 333 million WELLBY (156). The cost is likely far higher, as this assumes only 2 months of lockdown, and does not include the effect of loneliness on lifespan (i.e., early mortality) and disease that occurs particularly to young people (166–172).

The last cost considered here is the effect of unemployment. It is estimated that unemployment costs 0.7 WELLBY/unemployed person/year (145, 146). Since it is estimated there will be 400 million additional unemployment years due to the lockdowns, the cost is 280 million WELLBY/year (156, 173). The cost is likely at least 3X higher, as recovery from unemployment will occur over several years, we do not consider the effect on wellbeing to the families of the unemployed, and we do not consider the effect on deaths of despair in young people or on loss of health insurance.

The effects of loneliness and unemployment on life-expectancy are not considered in the costs above, only the loss of life-satisfaction in WELLBYs. Recent literature has summarized the major effect of individual income, social network index (i.e., integration in a social network), and adverse childhood experiences on life-span, early mortality, risk of chronic diseases (including heart disease, diabetes, kidney disease, stroke, cancer, lung disease, Alzheimer's, substance use, depression), and suicide rates (166–172). Recent financial difficulties, history of unemployment, lower life satisfaction, and history of food insecurity are associated with mortality in the United States (167). Actual or perceived social isolation is one of the top three risk factors for death due to cardiovascular disease, increases risk of death in the next decade by 25–30%, and “risks creating cohorts of individuals who are less socially functional (page 729)” (168, 174). Unemployment is associated with a mean adjusted hazard ratio for mortality of 1.63 (175). Life stress is associated with development and exacerbation of asthma, rheumatoid arthritis, anxiety disorders, depression, cardiovascular disease, chronic pain, HIV/AIDS, stroke, certain types of cancer, and premature mortality (176). Especially concerning are the effects on children during “the early years” of life, increasingly recognized as the period of greatest vulnerability to, and greatest return on investment from, preventing adverse long-term outcomes that can have lasting and profound impacts on future quality of life, education, earning potential, lifespan, and healthcare utilization (169–172). The early years of life are a critical period when a child's brain develops from social interaction and experiences, thus providing the foundation for their entire future life potential. During the pandemic children are being exposed to increased intimate partner violence, family financial crises, disrupted education, an increasing achievement gap (i.e., low-income families who do not have access to computer, internet, space, food, and parental support cannot participate in online learning), loneliness, physical inactivity, and lack of support services (e.g., school lunches, access to early childhood services and aids for those with disability) (87, 88, 104, 107, 177–179). These adverse childhood experiences have permanent impacts that cannot be compensated for by later improvements in social situations.

The cost-benefit analysis is shown in Table 6, finding on balance the lockdowns cost a minimum of 5X more WELLBY than they save, and more realistically, cost 50–87X more. Importantly, this cost does *not* include the collateral damage discussed above (from disrupted healthcare services, disrupted education, famine, social unrest, violence, and suicide) nor the major effect of loneliness and unemployment on lifespan and disease. Frijters and Krekel have estimated that “the [infection] fatality rate should be about 7.8% to break-even and make a radical containment and eradication policy worthwhile, presuming that would actually eliminate the disease (page 422)” (180). A similar cost-benefit analysis for Canada is shown in Supplementary Table 5, with the cost at least 10X higher for lockdowns than the benefit. A different analysis for Australia is shown in Table 7, estimating the minimum cost is 6.6X higher than the benefit of lockdown (181, 182). Another cost-benefit analysis for the UK used National Institute for Health and Care Excellence guidelines for resource decisions, that 1 QALY should cost no more than US$38.4K. Assuming lockdown could save up to 440 K people (although more likely at most: 66.65 million population × 40% to herd immunity × 0.24% IFR = 64 K people) of 5 QALY each, and a minimum GDP loss of 9% (i.e., assuming lost output comes back quickly, and not including any health costs of unemployment or disrupted education), “the economic costs of the lockdown… is far larger than annual total expenditure on the UK national health service… the benefits of that level of resources applied to health… would be expected to generate far more lives saved than is plausibly attributable to the lockdown in the UK… The cost per QALY saved of the lockdown looks to be far in excess… (often by a factor of 10 and more) of that considered acceptable for health treatments in the UK (page 9–10)” (147). The authors estimated the benefit of easing restrictions for over the next 3 months outweighs the cost by 7.3–14.6X (147). “A cost-benefit analysis of 5 extra days at COVID-19 alert level 4” for New Zealand found that the cost in QALY was 94.9X higher than the benefit (183). Finally, a cost-benefit analysis for the US is shown in Table 8, finding the cost of lockdown would be at least 5.2X the benefit (184, 185).

**Table 6:** Cost-Benefit analysis in WELLBYs for the world's response to COVID-19.

**Factor in World**	**Benefit**	**Cost**
COVID-19 deaths	360M WELLBY	–
Recession	–	1.2B WELLBY
Unemployment	–	280M WELLBY
Loneliness	–	333M WELLBY
Disrupted health services, disrupted education, famine, social unrest, violence, suicide	–	Not counted
TOTAL	360M WELLBY	1.813B WELLBY
**BALANCE**		**5X (minimum)−87X (maximum)**

*B, Billion; M, Million; WELLBY, wellbeing years. See text for details of the calculations*.

*Maximum: benefit reduced in half; recession effect increased 12X, unemployment effect increased 3X, and still not counting the disruption of health services, education, life-span effects of loneliness, etc*.

**Table 7:** Cost-benefit analysis in quality adjusted life years for Australia's response to COVID-19 (181, 182).

**Consideration**	**Cost/month**	**Benefit overall**	**Comment**
Wellbeing (immediate)	83,333 QALY	–	Attributes half of reduction (of 0.5 WELLBY) to lockdown
Reduced economic activity (government services)	25,812 QALY	–	Attributes half of yearly 6% loss in GDP to lockdown, and only government expenditure (not private) buys welfare (36% of GDP), at $100,000/QALY
Increased suicides	600 QALY	–	Expected to rise 25% over next 5 years, and attributes only 40% of this to lockdown
Disrupted non-university schooling	740 QALY	–	Foregone wages of children: each year of schooling yields approximately 9% more future earnings; assumes 80–90% equivalence of disrupted to normal school days
Disrupted health services, future mental stress and violence	–	–	Not included. Also does not consider bad habits inculcated (reduced physical activity, increased weight gain (for 40%), increased alcohol intake)
Reduced COVID-19 deaths		50,000 QALY	This is for lockdown “ad infinitum” (not per month); 0.04% of population saved
Total over 3 months of lockdown	331,485 QALY	50,000 QALY	Minimum cost is 6.6X any benefit

*QALY, Quality Adjusted Life Years; WELLBY, Wellbeing Years*.

**Table 8:** A cost-benefit analysis for lockdown in the US, modified from Cutler and Summer (184, 185).

**Factor**	**Quoted (184)**	**Revised**	**Explanation of revision**
**Cost**			
GDP loss	$7.592 Trillion	$7.592 Trillion^a^	No revision made. Note that, as the US accounts for 15% of world GDP, this translates to the global loss of $50.6 Trillion (as estimated in Table 6).
Mental health	0	$0.8 Trillion	Assuming that 50% of the mental health effect is from lockdowns
**Benefit**			
Deaths avoided	$4.4 Trillion	$0.3125 Trillion	Assuming the 625,000 deaths lose 5 QALY each at $100,000 per QALY. This is better than assuming each death, regardless of age or comorbidity, is the loss of the entire value of a statistical life. This is also how the cost on mental health was calculated.
Health impairment	$2.6 Trillion	$0.4875 Trillion	Assuming 35% of quality of life is lost *for the remaining years left* (likely 15 remaining years of 80 on average in a statistical life).
Mental Health	$1.6 Trillion	$0.8 Trillion	Assuming 50% of the mental health effects are due to not having lockdowns to prevent COVID-19 cases.
Cost-benefit balance	**Benefit 1.3X cost**	**Cost 5.2X benefit**	A minimal estimate: the GDP loss will likely be higher; willingness to pay for QALY is usually <$100,000/QALY, and NICE uses $30,000/QALY; not all deaths could be avoided by lockdown; at least 20% of excess deaths are not due to COVID-19 (i.e., are more likely from the response); severe cases (i.e., those that do not need intensive care, and may only need oxygen) likely have lower risk for health impairment of the severity modeled.

a *If the Value of a Statistical Life is accepted as used in the reference at $7 million, and the US economy will lose $7.592 Trillion in GDP over the decade, that is equivalent to the loss of 1,084,571 whole (statistical 80-year duration) lives = 86,765,680 years of lost life; that is equivalent to (assuming 5 QALY lost per COVID-19 death) 17,353,136 COVID-19 deaths*.

### Objection: The Economic Recession Would Happen Without Lockdown

This is unlikely, particularly if the fear is appropriately controlled with clear communication on risk, numbers with denominators and context, and important trade-offs, as this information becomes available. The resources and attention should be directed toward protecting the most vulnerable (i.e., older people, especially those with multiple co-morbidities). The evidence for policy impact on total human welfare should be based on a wide range of expertise, including economists, and not only health experts. The CIDRAP group published suggestions for communication during a crisis, which included advice to not over-reassure (i.e., be realistic about the course post-lockdown – cases and deaths will climb), to express uncertainty (i.e., explain the difficult dilemmas and trade-offs, and why we choose which course; explain that the initial reaction was temporary, buying time to figure out next steps); to validate emotions (i.e., admit waves of disease will occur and there may be economic devastation); and to admit and apologize for errors (i.e., we must resurrect a devastated economy in order to save lives) (186).

The severity of mandated lockdowns was directly linked with the severity of the economic collapse (147, 181, 187–191). These were direct commands to halt work, restrict travel, restrict the number of people inside dwellings, close factory floors, stay at home, etc. Economic activity, GDP loss, and unemployment were temporally, within weeks, related to lockdown orders (181). There was a dramatic decline in employment, consumer spending, and economic outcomes largely accounted for by different degrees of restrictions in different countries (181, 188, 189). The consensus, for example by the Bank of England, the Reserve Bank of Australia, the Organization for Economic Co-operation and Development, the International Monetary Fund (e.g., the “calamitous Great Lockdown”), and the Chief Medical Officer of Health in Canada (e.g., “the extensive slowdown in the Canadian economy as a result of public health emergency measures” (page 29), is that the economic recession is a result of the lockdowns (45, 117, 190–192).

### Objection: Consider the “Long-Haulers”

The long-term effects of COVID-19 illness need to be studied and clarified. Much of the current information is based on anecdotes (i.e., single cases) in the press. It may be expected that survivors of ARDS due to COVID-19 will have significant quality of life sequelae similar to ICU survivors from other causes of ARDS, or even lower given the lower cytokine levels in COVID-19 (193, 194). It may also be expected that some survivors of COVID-19 that did not require hospitalization will have significant lingering symptoms for months similar to what occurs with other causes of community acquired pneumonia (195). The few studies reported to date do not well quantify the severity and duration of long-term symptoms such as fatigue, breathlessness, “foggy thinking,” etc., making it difficult to interpret the impact on cost-benefit analyses (196–200). The highest rates of “long-COVID-19” are from crowdsourced online data where there is likely a strong selection bias in participation (201–203). In addition, most of these reports do not compare to contemporary controls during the pandemic, controls who are often experiencing social isolation, unemployment, and loneliness. For example, one survey of people without COVID-19 in the United States found a high prevalence of anxiety (25.5%), depressive (24.3%), and trauma and stressor related (26.3%) disorders, with 13.3% who started or increased substance use to cope, and 10.7% who seriously contemplated suicide in the last 30 days (204). The Household Pulse Survey in the found that in 2019 11% of adults had symptoms of anxiety or depressive disorder, while in April-August 2020 35–40% did (205). Another survey in US adults found the prevalence of depression symptoms was more than 3-fold higher during COVID-19 than before, and worse for those with lower social and economic resources (206). A survey in Australia found worse exercise (47.1%), mental wellbeing (41%), weight gain (38.9%), screen time (40–50%), and life satisfaction (down by an average of 13.9%) during the pandemic (207). In Canada, 57% of children 15–17 years old reported their mental health was “somewhat worse” or “much worse” than it was prior to physical distancing measures during the pandemic, and Canadians ≥15 years old had a 23% decrease in reported “excellent or very good self-perceived mental health” (177, 208). Although there will likely be many “long-haulers,” the incidence, severity, and duration of long-term symptoms would need to be very high to change the cost-benefit balance. Given that at a generous minimum the cost-benefit balance is at least 5X against lockdowns, the sequelae of COVID-19 would need to cost well over 200 million QALY worldwide, and likely >10X that number, to make the cost-benefit analysis in need of reconsideration.

### Objection: Low-Income Countries Are Particularly Susceptible and Need Protection

The Imperial College COVID-19 Response Team modeled the effect on low-income countries (209). These countries were hypothesized to be more susceptible to COVID-19 deaths, even with markedly lower population over age 65 years (about 3%), due to several factors: larger size of households (i.e., more homogeneous contact patterns), far fewer hospital and ICU beds, lower quality of health care, and unique co-morbidities (e.g., HIV in >1%, tuberculosis in >25%, and malnutrition in >30% of the population) (209). For suppression to have benefit, it was estimated to need to be in force 77% of the time (compared to 66% in high-income countries) over the 18 months of modeling [and “well beyond the time window of our simulations (page 421)”] (209). However, modeling inputs were overestimated, with >90% of the population infected, and baseline IFR in high-income countries 1.03%. Moreover, low-income countries are more vulnerable to lockdown adverse effects for several reasons: lower ability to work from home, more household based transmission (when confined to home), economic vulnerability (a higher degree of informal labor markets, and marginal capacity to provide support for ensuring livelihoods), slower build-up of herd immunity (given limited health care capacity), little testing capacity, wider health risks from diverting all attention to a single disease, and future health system failure once suppression measures are lifted (also see Table 1) (209, 210). The effects of a recession on government spending is magnified when this spending was already insufficient to improve the social determinants of health. In India, the desperation is leading to an increase in child trafficking (211). Surveys in Africa indicate a very low IFR; for example, in Kenyan blood donors 5% were seropositive yet the country reported only 100 deaths, in Bantyre, Malawi, a serosurvey found 12.3% of healthcare workers were seropositive yet only 17 deaths were reported, and in two cities in Mozambique seropositivity was 3 and 10% yet only 16 deaths were reported (212). It is extremely likely the cost-benefit analysis is even more against lockdown in low-income countries for these reasons.

## Discussion

### What to Do Now: Change the Trolley Track

#### Other Calls for a Change in Response Priorities

Several other groups and individuals have made calls for a change in COVID-19 response priorities (Table 9) (213–220). In an open letter on July 6, 2020, to the Prime Minister and Premiers of Canada signed by many former deputy ministers of health, chief public health officers, and medical deans, the authors called for “A Balanced Response” (213). They write that the current approach “carries significant risks to overall population health and threatens to increase inequalities… Aiming to prevent or contain every case of COVID-19 is simply no longer sustainable…” (213). In an open letter to the National Cabinet in Australia signed by many economists and medical experts with the Australian Institute for Progress, the authors make similar points (214). They write that “to analyze the COVID-19 effect it is necessary to understand it as shortening life. But the lockdowns and the panic have also had a cost in shortening life for others” (214). Ioannidis called for evidence to guide policy, noting many of the collateral and recession effects discussed above (215–219). “Shutdowns are an extreme measure. We know very well that they cause tremendous harm” (216). A resignation letter by an economist in the Australian Treasury wrote that “the pandemic policies being pursued in Australia… are having hugely adverse economic, social and health effects… The need for good policy process does not disappear just because we face a public health crisis…” (220). The “Great Barrington Declaration” written on October 4, 2020, by infectious disease epidemiologists and public health scientists recommends “Focused Protection” (221). The declaration writes that “current lockdown policies are producing devastating effects on short and long-term public health… leading to greater excess mortality in years to come…” (221).

**Table 9:** Other calls for a change in COVID-19 response priorities.

**References**	**Content of the call for adjusting COVID-19 response priorities**
Open letter on July 6, 2020, to the Prime Minister and Premiers of Canada (213)	The current approach “carries significant risks to overall population health and threatens to increase inequalities… Aiming to prevent or contain every case of COVID-19 is simply no longer sustainable… We need to accept that COVID-19 will be with us for some time and to find ways to deal with it.”
	The response risks “significantly harming our children, particularly the very young, by affecting their development, with life-long consequences in terms of education, skills development, income and overall health.”
	Suggest that we need “to focus on preventing deaths and serious illness by protecting the vulnerable while enabling society to function and thrive… While there is hope for a vaccine to be developed soon, we must be realistic about the time… We need to accept that there will be cases and outbreaks of COVID-19.”
	“Canadians have developed a fear of COVID-19. Going forward they have to be supported in understanding their true level of risk… while getting on with their lives – back to work, back to school, back to healthy lives and vibrant, active communities….”
	COVID-19 “is not the only nor the most important challenge to the health of people in Canada… The fundamental determinants of health – education, employment, social connection and medical and dental care – must take priority…”
Open letter to National Cabinet of Australia (214)	“exposure to COVID-19 is only temporarily avoidable”; “to analyze the COVID-19 effect it is necessary to understand it as shortening life. But the lockdowns and the panic have also had a cost in shortening life for others.”
	Some of these costs include that the lockdown: “will decrease national income… and this will have a measurable effect on the length of the average lifespan,” “[has] disrupted normal health services… estimated an increase in cancer deaths over the next 12 months of 20%,” [and will cause] future suicides by the unemployed and others whose lives have been ruined.”
	Urge for “a cost-benefit analysis, including lives saved versus lives lost, both directly and consequentially… [and] weekly or daily non-epidemic death figures should be posted as well as deaths from the epidemic…”
Ioannidis, JPA (95, 215–219)	Called for evidence to guide policy, noting many of the collateral and recession effects discussed above.
	“Shutdowns are an extreme measure. We know very well that they cause tremendous harm.”
	“the excess deaths from the measures taken is likely to be much larger than the COVID-19 deaths… learning to live with COVID-19 and using effective, precise, least disruptive measures is essential to avoid such disasters and to help minimize the adverse impact of the pandemic” (95).
	“When major decisions (e.g., draconian lockdowns) are based on forecasts, the harms (in terms of health, economy, and society at large) and the asymmetry of risks need to be approached in a holistic fashion, considering the totality of the evidence” (219).
Resignation letter by economist in Victorian Treasury (220)	“the pandemic policies being pursued in Australia… are having hugely adverse economic, social and health effects… The need for good policy process does not disappear just because we face a public health crisis… the elderly are many times more vulnerable to a serious outcome than the young. It was necessary, therefore, to work out a targeted age-based strategy… The direct and indirect costs imposed by regulatory approaches may not be… immediately obvious. Risk regulation that is poorly targeted or costly will divert resources from other priorities… needed to commission a cost-benefit analysis of alternative policy options….”
	Governments should have realized “they are hostage to chronic groupthink and actively sought alternative advice… instead of performing its taxpayer-funded duty of providing forthright analysis of alternatives… can (even now) be managed by isolating the elderly and taking a range of voluntary, innovative measures.”
The Great Barrington Declaration (221)	“current lockdown policies are producing devastating effects on short and long-term public health… leading to greater excess mortality in years to come… keeping students out of school is a grave injustice… The most compassionate approach that balances the risks and benefits of reaching herd immunity, is to allow those who are at minimal risk of death to live their lives normally to build up immunity to the virus through natural infection, while better protecting those who are at highest risk.”

A caveat to quoting these open letters is that “petitions should not be used to prove that the positions of the signatories are scientifically correct (page 1),” as this would be based on the fallacies of “argument ad populum” and “invoking authority,” and have other drawbacks (222). These open letters are used only to show that many have expressed views similar to those expressed here, and this might open the door to serious consideration of the empirical evidence and arguments presented above.

#### Objection: Herd Immunity Is a Dangerous Idea

There are several objections that have been made to the idea of opening up society to achieve natural herd immunity (223–226).

First, an objection is that natural herd immunity assumes the immunity is long lasting, and this may not be the case (223–226). If immunity is short-lived, then COVID-19 may become an endemic and likely yearly viral infection as predicted by Kissler et al. (2). In the event of short-lived immunity it may still be important to achieve natural herd immunity to protect the high-risk groups (i.e., older people) now and yearly (until a vaccine is widely available) without recurrent and prolonged lockdowns that devastate the economy and thus population life-expectancy and wellbeing. Notably, if immunity is not long-lasting this will be a problem for possible vaccine induced herd immunity as well, as the world population will need vaccines to be produced and delivered everywhere at least each year.

Second, another objection is that the costs in deaths, mental and physical health and suffering, socioeconomic inequities, and harming the economy will be too high (223, 224). This objection ignores the discussion above of the trade-offs involved that include not only COVID-19 direct effects, but also indirect effects of the response to COVID-19, the collateral damage and cost-benefit analysis where it was shown that the costs of all these effects is in fact much higher with lockdowns.

Third is the objection that uncontrolled transmission in younger people would inevitably result in infections in high-risk groups with high mortality (223–226). The ability to successfully shield continuing care facilities and hospitals from COVID-19 is questioned (223, 224). Prolonged isolation of high-risk groups is said to be “unethical (page e71)” (223). The objection is odd, as if we cannot protect those in nursing homes nor hospitals, why are we using personal protective equipment at all? In addition, prolonged isolation of *all* groups is what has occurred now, and based on the cost-benefit analysis this is what is unethical by causing far more harm to all, including the high-risk older population. Of course, infection *can* still spread to high-mortality populations; however, the goal is to reduce this risk. Moreover, <10% of the population is at high-risk, accounting for >90% of potential deaths; surely we can focus on protecting this subgroup of people (219). Early monitoring in Europe shows that despite increasing COVID-19 cases, excess mortality has only shown a slight increase, suggesting protection of the most vulnerable may be feasible (227). Modeling has also suggested that social distancing of those over 70 years of age would prevent more deaths than a fixed duration of social distancing of the entire population (228).

Fourth is the objection that healthcare systems will be overwhelmed by uncontrolled spread (223, 224). This is a worrisome possibility, as health-care providers may be forced to make painful rationing decisions. If a healthcare system is overwhelmed, the effects would have to be extreme to make the benefit of lockdowns to save ICU capacity comparable to the long-term costs. There are several ways to minimize this possibility, including a focus on protecting those at high-risk (see below), information dissemination to cause fast awareness of voluntary sensible self-imposed use of handwashing and (in crowded areas) masks (229, 230), limiting very large gatherings, and expanding critical care capacity when necessary. Forecasting of healthcare capacity needs in the short or medium term, even when built directly on data and for next day predictions, has consistently failed, and most healthcare systems were not overwhelmed despite sometimes being stressed with high peaks of cases (219, 231). Forecasting failure led to frail older patients being discharged to nursing homes (where there was high mortality), and largely empty wards (unnecessarily affecting hospital utilization for other serious conditions); in Canada “overall ICU occupancy rates did not exceed 65% (page 12)” (45, 219). Lockdowns in anticipation of forecast healthcare incapacity should not be done, especially if based on forecasting that is not released for public scrutiny nor repeatedly fit to real-time data to verify accuracy. In addition, if there are insufficient ICU beds for the population due to underfunding, the effects of the recession on government healthcare spending in the future will markedly adversely worsen this situation in the long-term.

Fifth is the objection that natural herd immunity is not achievable (223–226). This is based on the few case reports of re-infection, the Brazilian city of Manaus where adjusted seroprevalence was up to 66% yet there is currently a resurgence of COVID-19 cases, and the claim that natural herd-immunity has never occurred. The seven published case reports of re-infection, four with symptoms (one requiring hospitalization, and one death in an immunocompromised 89 year-old with few details reported), when 10% of the world population has likely been infected over the past 10 months cannot yet provide evidence that severe reinfection and contagion is at all common (232–237). This also applies to a more recent review finding 17 cases of reinfection reported of whom 3 were hospitalized and the one died (238). Regarding Manaus, the high seroprevalence likely reflected the special situation of a relatively homogeneous cohort of people in overcrowded low socioeconomic urban situations, with reliance on crowded long riverboat travel; now there seems to be a different demographic cohort of young wealthy individuals being exposed (239–241). The resurgence of cases in Manaus was described as follows: “from the second week of August there has been a small increase in the number of cases which, at the time of writing, has begun to decline (page 3)” (241). In addition, the peak seroprevalence in blood donors in Manaus was 51.8% in June, while another study of household seroprevalence in Manaus on May 14–21 found this to be 12.7% (the respective numbers for São Paulo were closer, at 6.9 and 3.3% in the two serosurveys) (241, 242). Even correcting for a possible lower sensitivity of capillary blood used in the household survey does not explain the difference, as the corrected seroprevalence might be up to 19.3% (243). Regarding historical natural herd-immunity, it is likely that this was achieved for several infections, with outbreaks that occurred as births added sufficient numbers of new susceptible young individuals (e.g., for Measles, Mumps, Rubella, Pertussis, Chickenpox, Polio) (244, 245). The current vaccines are given intramuscular, and thus do not induce mucosal IgA responses thought necessary to prevent upper respiratory tract SARS-CoV-2 infection (246). These vaccines have unknown efficacy on infection and infectiousness of SARS-CoV-2 in those protected from COVID-19 symptoms (246–248). Herd immunity is premised on the efficacy of immunity in preventing transmission (244), and it is possible that current vaccines will have little effect on this.

Finally, an important point to emphasize is that the information in this review does *not* depend on natural herd immunity being achieved. The collateral damage, and the cost-benefit analysis showed that lockdowns are far more harmful than a risk-tailored population specific response. “Public health is the science and action of promoting health, preventing disease, and prolonging life… ensuring that Canadians can live healthy and happier lives (page 59–60)” (45); some suggestions for how to do this are discussed below.

#### Some Suggestions: What Can We Do?

##### Focus on Protecting Those at High Risk

A risk-tailored, population-specific response (249). This starts with better public understanding of the risks and trade-offs involved (186). Protection should focus on high-risk groups: those hospitalized (e.g., prevent nosocomial infection) (216), in nursing homes (e.g., staff work in only one facility, adequate personal protective equipment supply, more staff, equitable pay) (250), prisons, homeless shelters, and certain demographics (e.g., age ≥ 70 years, especially those with multiple severe co-morbidities) (249). There should be investment in improving the social determinants of health [e.g., “invest in strategies that address health inequities and better serve the elderly, people experiencing homelessness, and those living with limited means (page E685)” (249)] (45, 160, 251). That this is so important is demonstrated by the sobering fact that “Black disadvantage operates every year on the scale [in terms of age-adjusted mortality and life-expectancy in the United States] of White's experience with COVID-19 (page 21854)” (252). Do not lock everyone down, regardless of their individual risk, as this will cause more harm than benefit (216). It is not true that “no one is protected until everyone is protected (page 2)” (45).

##### Open Schools for Children (87, 253)

School provides essential educational, social, and developmental benefits to children (254). Children have very low morbidity and mortality from COVID-19 (174), and, especially those ≤10 years old, are less likely to be infected by SARS-CoV-2 (57, 255–258), and have a low likelihood to be the source of transmission of SARS-CoV-2 (178, 258). Children account for 1.9% of confirmed cases worldwide (259). School closures don't seem to have an impact on community outbreaks (178, 260). Modeling predicted that school and university closures and isolation of younger people would increase the total number of deaths (postponed to a second and subsequent waves) (228). Modeling also predicted that school closures alone would prevent only 2–4% of deaths (261). The infection mortality risk from influenza is higher than from COVID-19 for people age <50-years, and about 2.9X lower for those 50–64-years (still, 99.86% survived SARS-CoV-2 infection) (see Supplementary Table 6) (41, 262, 263). We need to educate parents and teachers regarding their low risk, and focus teachers with greater vulnerability due to age and multiple co-morbidity on remote learning. Until schools open, education is lacking especially for those with the fewest opportunities, worsening social disparities that education systems are intended to level. Similarly, allow visitation in children's hospitals and pediatric long-term care facilities, where the risk even with co-morbidities is so low as to not warrant the tragedy of sacrificing our most vulnerable in the false hope of protecting them (43, 48, 49, 178).

##### Consider Increasing Health Care Surge Capacity

If forecasting, accurately calibrated repeatedly to real-time data [up to now, forecasting, even short-term, has repeatedly failed (219, 231)], suggests it is needed, health care surge capacity should be increased. With universal masking in hospitals, asymptomatic health care workers can continue to work as their transmission risk, even if infected, is very low, and this can preserve staff coverage (264, 265).

##### Build Back Better

Maybe we have learned that the “government can intervene decisively once the scale of an emergency is [or seems] clear and public support is present (page 4)” (266). Maybe we can “recalibrate our sense of omnipotence” seeing “the ability of ‘natural’ forces to shock the global economy” (page 4) (266). Maybe we can tip “energy and industrial systems toward newer, cleaner, and ultimately cheaper modes of production that become impossible to outcompete (page 4)” (266). This would involve investment in clean technologies (e.g., renewable energy, green construction, natural capital, carbon capture and storage technologies), and conditional (on measurable transition) bailouts. This is because climate change, like the COVID-19 response, will involve market failures, externalities, international cooperation, and political leadership: the devastation is just in slow motion and far graver. The aggregate fiscal stimuli aimed at alleviating the consequences of the COVID-19 crisis for 149 countries amount to US$12.2 trillion (267). Climate experts have estimated that “the additional investment needed to shift low-carbon energy investment onto a Paris-compatible pathway thus amounts to about US$300 billion per year globally over the coming 5 years… 12% [of total pledged stimulus to date] when considered over the entire 2020-2024 period… (page 299)” (267). Moreover, “subtracting divestments from high-carbon fossil fuels… indicates that the overall increase in net annual investments to achieve an ambitious low-carbon transformation in the energy sector are notably small… 1% [of the total announced stimulus to date] over the 2020-2024 period (page 299)” (267). A green recovery may be a driver of employment, spur innovation and diffusion of technologies, reduce stranded assets, and result in a more sustainable and resilient society (117, 267).

### Some Research Priorities

More information will help to optimize responses to the pandemic. This particularly applies to possible prevention, prophylaxis, and treatment of COVID-19. How effective cloth masks are at preventing infection, or at reducing severity of infection needs more study (268, 269). The safety, efficacy (including for interrupting infectiousness, necessary to achieve herd immunity), and durability of protection from vaccines, particularly in high-risk groups, must be determined in large Phase III randomized controlled trials (246). Novel treatments are in clinical trials, with dexamethasone having benefit on mortality in those with severe COVID-19 requiring oxygen treatment (270). Research is also required to determine the frequency and severity of reinfections (271). The frequency, duration, and severity of “long-COVID” requires better study. The impact of influenza on COVID-19 morbidity and mortality requires study, as both viruses may compete for the same susceptible individuals (271). Importantly, research on “the impending authoritarian pandemic… [the] toll being inflicted on democracy, civil liberties, fundamental freedoms, [and] healthcare ethics… (page 1)” [e.g., due to those responses that were not strictly necessary nor proportionate, largely copied from the “authoritarian example of others (page 5)”] is required to prevent regression and “erosion of rights-protective democratic ideals and institutions (page 1)” (272) across the globe (272–275).

## Conclusion

The lockdowns implemented in the name of public health entailed trade-offs that were not adequately considered (275). Lockdowns may prevent some COVID-19 deaths by flattening the curve of cases and preventing stress on hospitals. At the same time, lockdowns cause severe adverse effects for many millions of people, disproportionately for those already disadvantaged among us. The collateral damage included severe losses to current and future wellbeing from unemployment, poverty, food insecurity, interrupted preventive, diagnostic, and therapeutic healthcare, interrupted education, loneliness and deterioration of mental health, and intimate partner violence. The economic recession has been framed as the economy vs. saving lives from COVID-19, but this is a false dichotomy. The economic recession, through austerity in government spending on the social determinants of health, can be expected to cause far more loss of life and wellbeing over the long-run than COVID-19 can. We must open up society to save many more lives than we can by attempting to avoid every case (or even most cases) of COVID-19. It is past time to take an effortful pause, calibrate our response to the true risk, make rational cost-benefit analyses of the trade-offs, and end the lockdown groupthink.

## Author Contributions

AJ wrote the manuscript, and approved the final version.

## Conflict of Interest

Theauthor declares that the research was conducted in the absence of any commercial or financial relationships that could be construed as a potential conflict of interest.

## Abbreviations

COVID-19: Coronavirus Disease 2019
GDP: Gross Domestic Product
IFR: Infection Fatality Rate
ICU: Intensive Care Unit
NPI: Non-pharmaceutical Intervention
QALY: Quality Adjusted Life Years
SARS-CoV-2: Severe Acute Respiratory Syndrome Coronavirus 2
UK: United Kingdom
US: United States
WELLBY: Wellbeing Adjusted Life Years.
