# Supplementary material for: COVID-19: Rethinking the Lockdown Groupthink
Source: Front Public Health. 2021 Feb 26;9:625778. doi: 10.3389/fpubh.2021.625778 (PMC7952324; doi:10.3389/fpubh.2021.625778)
Supplement: Supplementary file 1 [file Data_Sheet_1.PDF]

ETable 1. Total and COVID-19 deaths in the USA, as of August 22, 2020.

**Age group**	**COVID deaths in 6 months to Aug 22**	**Deaths from all causes to Aug 22**	**COVID as % of deaths in 2020**
0-14	57	14679	**0.39%**
15-24	280	18594	**1.51%**
25-44	4558	93066	**4.90%**
45-54	8648	100926	**8.57%**
55-64	20655	231983	**8.90%**
65-74	34980	351806	**9.94%**
75-84	43392	430582	**10.08%**
85+	51710	537185	**9.63%**
**TOTAL**	**164280**	**1778821**	**9.24%**

Assumes all deaths *with* COVID-19 are deaths *from* COVID-19.
Reference: 123

ETable 2. COVID-19 deaths in Canada as of August 30, 2020 compared to deaths in 2018.

Age group	COVID deaths in 6 months of 2020	Deaths in all of 2018	COVID as % of deaths over 6 months of 2020
0-19	1	3092	**0.06%**
20-29	9	3273	**0.55%**
30-39	15	4455	**0.67%**
40-49	50	7287	**1.35%**
50-59	211	19959	**2.07%**
60-69	651	40231	**3.13%**
70-79	1635	60143	**5.16%**
80+	6420	146266	**8.07%**
**TOTAL**	**8992**	**283706**	**5.96%**

In 2018 there were 23642 deaths/month and 777 deaths/day in Canada.
References: 124, 125

ETable 3. COVID-19 deaths globally as of late November, 2020 compared to deaths in 2018-2019.

Region	Deaths with COVID-19 to Nov 21 (or 15), 2020	Deaths in 2018 or (2019)	X 75% of deaths in 2018 or (2019)	% of deaths with COVID-19
**Global**	1,338,100	(58,394,000)	(43,795,500)	**3.06%**
**EU**	(341,488)	(8,285,000)	(6,213,750)	**5.50%**
**Africa**	(31,450)	(10,434,000)	(7,825,500)	**0.40%**
**Asia**	(243,912)	(32,053,000)	(24,039,750)	**1.01%**
**Americas**	(675,735)	(7,337,000)	(5,502,750)	**12.28%**
**Canada**	11,406	(291,000)	(218,250)	**5.23%**
**Alberta**	471	25,990	19,493	**2.42%**
**Ontario**	3,472	106,991	80,243	**4.33%**
**Quebec**	6,806	67,216	50,412	**13.50%**
**USA**	256,314	(2,909,000)	(2,181,750)	**11.75%**
**Brazil**	168,989	(1,377,000)	(1,032,750)	**16.36%**
**Oceania**	(16,377)	(285,000)	(213,750)	**7.66%**

EU: European Union

References: 123, 125, 127, 128

ETable 4. Studies suggesting that efficacy of nonpharmaceutical interventions to prevent spread of COVID-19 are not as high as some predicted.

Study	Details of efficacy of non-pharmaceutical intervention
Luskin DL[149]	Using “highly detailed anonymized cellphone tracking data provided by Google… tabulated by the University of Maryland’s Transportation Institute into a ‘social distancing index’”, it was found that lockdown severity correlated with a greater spread of the virus, even when excluding states with the heaviest caseloads, and not with population density, age, ethnicity, prevalence of nursing homes, or general health, suggesting that “[heavy] lockdowns probably didn’t help.”
	This analysis also found that states that subsequently opened-up the most tended to have the lightest caseloads, suggesting that “opening up [a lot] didn’t hurt.”
Atkeson A, et al.[150]	An analysis across 23 countries and 25 states each with >1000 deaths by July 22 found that the growth rates of daily deaths from COVID-19 fell rapidly [from a wide range of initially high levels - doubling every 2-3 days] within the first 30 days after each region reached 25 cumulative deaths, and has hovered around zero or slightly below since.
	Epidemiological models found that this implied both the Re and transmission rates fell rapidly from widely dispersed initial levels [Re≥3], and the Re has hovered around 1 after the first 30 days of the epidemic virtually everywhere in the world.
	The authors suggest that there must be “an omitted variable bias” accounting for this finding [and similar findings in previous pandemics], that the role of region-specific NPI’s implemented in the early phase of the pandemic is likely overstated, and that the removal of lockdown policies has had little effect on transmission rates.
Chaudhry R, et al.[151]	A study using data from the top 50 countries ranked by number of cases found that “rapid border closures, full lockdowns, and wide-spread testing were not associated with COVID-19 mortality per million people.”
Wood SN[152]	A mathematical model using “a Bayesian inverse problem approach applied to UK data on COVID-19 deaths and the disease duration distribution” suggested that “infections were in decline before the full UK lockdown (March 24), and that infections in Sweden started to decline only a day or two later.”
Chin V, et al.[153]	The model for Europe used in [7] was based on circular reasoning [i.e., having modelled Re “as a step function and only allowed to change in response to an intervention”]. Using a model allowing for gradual changes over time and better fitting the data, complete lockdown had “no or little effect, since it was introduced typically at a point when Rt was already low.” For example, when lockdown was adopted in the UK, “Rt had already decreased to 1.46.” In fact, “lockdown and event ban had similar effect sizes on the reduction of Rt”. Overall, “one cannot exclude that the attribution of benefit to complete lockdown is a modelling artefact.”
Homburg S, Kuhbandner C.[154]	The model in [7] used circular reasoning [“the purported effects are pure artefacts”] by “using as an a priori restriction that Rt may only change at those dates where interventions become effective.” In the UK “the growth factor had already declined… strongly suggests that the UK lockdown was both superfluous… and ineffective.” In addition, the attribution of the decline in Sweden’s Rt to banning of public events is odd because that was an “NPI that they found ineffective in all other countries.”
Islam N, et al.[155]	Implementation of any physical distancing intervention [including lockdown] was associated with an overall reduction in COVID-19 incidence of only 13% [IRR 0.87, 95% CI 0.85 to 0.89] in 149 countries. There was no effect on this estimate of days since the first reported case of COVID-19 until the first implementation of physical distancing policies.

ETable 5. Cost-benefit analysis in WELLBYs for Canada's response to COVID-19

Factor in Canada	Benefit per month	Cost per month
COVID-19 deaths	37.59M X 0.5 for herd X 0.003 IFR X 5 QALY/ 12 months = 23,494 QALY = **140,963 WELLBY**	-
Recession	-	(1.713T GDP/12 months X 0.15 GDP loss X 0.4 government spending)/100K = 85,650 QALY = **513,900 WELLBY**
Unemployment	-	2M X 0.7/12 months = **116,667 WELLBY**
Loneliness (if we end half of lockdown)	-	37.59M/2 X 0.5/12 months = **783,125 WELLBY**
Disrupted health services, disrupted education	-	Not counted
TOTAL	**0.141M WELLBY**	**1.41M WELLBY**
**BALANCE**		**10X [minimum]**

IFR: infection fatality rate; K: thousands; M: Million; QALY: quality adjusted life years; WELLBY: wellbeing years

ETable 6. Infection fatality rate for Influenza in the United States and for SARS-CoV-2 in Switzerland by age group.

	**Influenza in the United States**[262]				**COVID-19 in Geneva**[41]
**Age group (years)**	**Illness rate/100,000 2017-18 (cases 2018-19)**	**Mortality rate/100,000 2017-18 (cases 2018-19)**	**CFR % 2017-18 2018-19**	**IFR % (assuming 20% asymptomatic)**[263]	**IFR %**
0-4	18448.1 (3633104)	0.6 (266)	0.0033 (0.0073)	0.0026 (0.0059)	-
5-17	13985.6 (7663310)	1.0 (211)	0.0072 (0.0028)	0.0057 (0.0022)	<0.0016
18-49	10469.7 (11913203)	2.0 (2450)	0.019 (0.021)	0.015 (0.016)	0.0092
50-64	20881.1 (9238038)	10.6 (5676)	0.051 (0.061)	0.041 (0.049)	0.14
<65	(32447655)	(8603)	(0.027)	(0.021)	median 0.05[26]
65+	11690.6 (3073227)	100.1 (25555)	0.86 (0.83)	0.68 (0.67)	2.7

The median IFR for those <65 years for COVID-19 was assumed to be the same as for those <70 years in reference 26.
